# Adherence to Methotrexate in Rheumatoid Arthritis: A Danish Nationwide Cohort Study

**DOI:** 10.1155/2015/915142

**Published:** 2015-02-25

**Authors:** Henning Bliddal, Stine A. Eriksen, Robin Christensen, Tove Lorenzen, Michael S. Hansen, Mikkel Østergaard, Lene Dreyer, George Luta, Peter Vestergaard

**Affiliations:** ^1^The Parker Institute, Department of Rheumatology, Copenhagen University Hospital at Bispebjerg and Frederiksberg, 2000 Copenhagen F, Denmark; ^2^Department of Health Science and Technology, Center for Sensory-Motor Interaction (SMI), Aalborg University, 9220 Aalborg, Denmark; ^3^Aalborg University Hospital, Clinical Institute, 9100 Aalborg, Denmark; ^4^Department of Rheumatology, Region Hospital, 8600 Silkeborg, Denmark; ^5^Clinic of Rheumatology, 4000 Roskilde, Denmark; ^6^Copenhagen Centre for Arthritis Research, Center for Rheumatology and Spine Diseases, Glostrup Hospital, 2600 Glostrup, Denmark; ^7^Department of Rheumatology, Copenhagen University Hospital, 2900 Gentofte, Denmark; ^8^Department of Biostatistics, Bioinformatics, and Biomathematics, Georgetown University, Washington, DC 20057, USA; ^9^Department of Endocrinology, Aalborg University Hospital, Aalborg, Denmark

## Abstract

*Objectives*. To study adherence to methotrexate (MTX) and factors of importance thereof in patients with rheumatoid arthritis (RA).* Methods*. Patients with a hospital diagnosis of RA (ICD10 codes M05.X or M06.X) after January 1, 1997, and aged ≥18 years at the date of first diagnosis/contact, with at least one prescription of MTX (L04AX03), were included.* Results*. A total of 18,703 (47.6%) patients had ever used MTX among 39,286 with a diagnosis of RA; among the MTX users, 16,503 (88.2%) had filed more than one MTX prescription. The median time from diagnosis to first MTX prescription was 0.66 (IQR 0.26–1.80) years. In those who filed more than one MTX prescription, the mean adherence time for ≥7.5 mg MTX per week was 1,925 (IQR 467–3,056) days for patients treated in private practice versus 1,892 (IQR 452–3,316) days for patients treated in hospital. The main determinants of nonadherence were female gender, younger age, and time from diagnosis to initiation of MTX.* Conclusions*. Treatment at hospital or in private practice did not influence the adherence to MTX. Nonmodifiable factors of importance were gender and age, while adherence to MTX therapy decreased with time lapse between diagnosis and prescription.

## 1. Introduction

The diagnosis “rheumatoid arthritis” (RA) is based on a pattern of symptoms and physical examination findings, with and often without any radiographic or serologic abnormalities [1, 2]. To avoid joint damage, inflammation in patients with RA should be suppressed as much as possible [3]. According to the treat-to-target paradigm, the primary goal of treating the RA patient is to maximise long-term health-related quality of life through control of symptoms, prevention of structural damage, normalisation of function, and social participation [4, 5]. Methotrexate (MTX) is the appropriate first-line agent for most patients with RA [5]; MTX is the “anchor drug” for treatment of RA. This is due to a combination of effectiveness and a favourable safety profile compared to other disease-modifying antirheumatic drugs (DMARDs) [5, 6].

Real-life registry data indicate that patients to a larger extent remain on antirheumatic biologic agents when given in combination with MTX [7]. However, preliminary Canadian healthcare data indicate that when MTX is prescribed in conjunction with a biologic agent, more than half of the patients do not collect their MTX prescription [8, 9]. If these estimates are justified, the clinical practice guidelines could be in conflict with the item accepted unanimously by the “treat-to-target” task force stating that “*the treatment of RA must be based on a shared decision between patient and rheumatologist*” [4]. Adherence to MTX may fluctuate; that is, patients do no quit their medication fully but possibly taper the intake over time. Based on this assumption, the current study used a definition of adherence with cut-offs corresponding to use of MTX at or above 7.5 mg (3 tablets of 2.5 mg) per week, respectively, 5 mg per week.

The objective of this population based cohort study was to explore the adherence to MTX use in the Danish population in subjects with a hospital registered diagnosis of rheumatoid arthritis.

## 2. Methods

### 2.1. Study Design

The study was designed as a cohort study with patients with a hospital diagnosis of arthritis (ICD10 codes M05.X, M06.X). The cohort was constructed using data from (1) the* National Hospital Discharge Register *(Landspatientregisteret—an ICD10 diagnosis of M05.X or M06.X) and (2) the* National Prescription Register *(Lægemiddeldatabasen—at least one prescription of MTX) for the period January 1, 1997, until December 31, 2012 (both dates included). In order to exclude prevalent cases, only subjects diagnosed after January 1, 1998, and aged ≥18 years at the date of first diagnosis/contact and who had filed at least one prescription for MTX (ATC L04AX03) were considered. The index date was the date of first MTX prescription on or after January 1, 1998 (and after the first ICD10 M05X or M06X diagnosis). The cohort was constructed from the Danish population of approximately 5.4 million inhabitants. The patients were followed until death, migration, December 31, 2012, or any specified endpoint.

### 2.2. Registers Used

The information on occurrence of specified diseases is registered in the National Hospital Discharge Register founded in 1977 [10]. It covers all inpatient contacts from 1977 to 1994 and from 1995 also all outpatient visits to hospitals, outpatient clinics, and emergency rooms. Upon discharge, the physician codes the contact diagnosis using the ICD system. The code used is at the discretion of the individual physician. The register has a nationwide coverage and an almost 100% capture of contacts. In general the validity of registrations is high [11].

The Danish Medicines Agency keeps a nationwide register of all drugs sold at pharmacies throughout the country from 1996 and onwards (The National Pharmacological Database run by the Danish Medicines Agency: http://www.dkma.dk). Any drugs bought are registered with an ATC code, dosage sold, form of administration (tablets, injections, etc.), and date of sale. Information on patient contacts to practising specialists (here rheumatologists) was obtained from the National Register on contacts to general practitioners or specialists (Sygesikringsregistret). This register is used to reimburse, for example, specialists based on the consultations or treatments performed. Information on vital status and migrations was extracted from the National Person Register. All subjects are followed up until time of death, migration, any defined event, or December 31, 2012, whichever came first.

The present study linked these sources of information through the Central Person Register Number which is a unique registration code given to every inhabitant—to some degree similar to the American social security number—that allows registration on an individual basis but under code for the researchers who had no specific access to individualized data. The project was approved by the National Board of Health and the Danish Data Protection Agency.

### 2.3. Outcomes and Potential Endpoint Predictors

It was hypothesised that MTX adherence is better in RA patients treated by private practising rheumatologists compared to RA patients treated in hospital outpatient clinics* and* MTX adherence diminishes over time with concomitant use of prednisolone.

*Outcomes*. Cessation of MTX use is defined as (1) filed prescriptions of less than 7.5 mg MTX per week of observation or (2) filed prescriptions of less than 5 mg MTX per week of observation. In this instance it should be noted that 7.5 mg MTX per week is the recommended minimal dose to attain remission.

*Exposure Variables.* They are use of NSAIDs within the last year prior to diagnosis, use of prednisone/prednisolone within the last year prior to diagnosis, duration of RA diagnosis before MTX prescription, and the fact of being seen by a practising specialist in rheumatology in private practice versus being managed in hospital.

*Confounders/Effect Modifiers*. They are age, gender, and Charlson comorbidity index (except for connective tissue disorders, which were covered by the inclusion criteria) [12]. The Charlson index was chosen as this is a generally accepted register based evaluation of comorbidity.

### 2.4. Statistical Analysis

Mean and standard deviations (SD) as well as frequencies were used as descriptive statistics. Adherence to MTX was analysed using Kaplan-Meier survival functions. Analyses were performed using *t*-test for two samples, *χ* ^2^-statistics, and Cox proportional hazard regression as appropriate. In the Cox regression the proportional hazard assumption was tested using log-minus-log plots. A significance level of 0.05 was chosen. SPSS20.0 (IBM Corp.) and STATA12 (STATA Corp.) were used for the analyses.

## 3. Results

### 3.1. Participants

**Figure 1:**
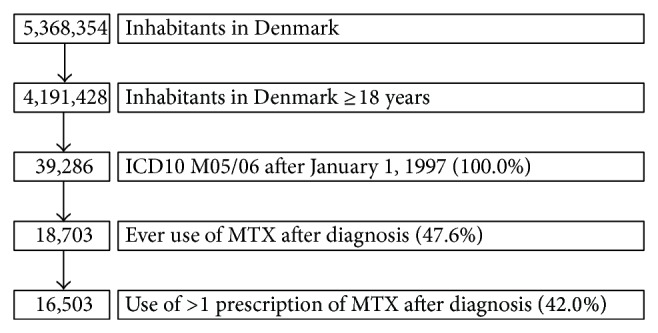
Study population (flow diagram). The study period began January 1, 1997. However, to exclude prevalent cases, only those with a de novo diagnosis after January 1, 1998, were included.

Figure 1 shows the sampling profile among inhabitants in Denmark: from the 5.4 million eligible inhabitants, 4.1 million were aged 18 years or more, and of these 39,286 had had a diagnosis of RA, and among these a little less than half of them (18,703; 47.6%) had ever filed a prescription for MTX. Due to the extensive nature of the registers, all could be followed until time of death, migration, or December 31, 2012, whichever came first.  Table 1 shows the characteristics of the participants and information on exposures and potential confounders. Comorbid conditions were scarce—most numbering a few per cent.

### 3.2. Main Results

The patients were followed up for a total of 146,012 years (mean: 7.8 ± 3.7 years, median 7.7 years). Long-term follow-up was thus present in most patients. Out of the 18,703, a total of 6,160 (32.9%) took less than 5 mg of MTX per week of follow-up, while 8,131 (43.5%) took less than 7.5 mg of MTX per week of follow-up. By consequence, many RA patients took less than the lowest recommended dose of MTX. The median time from diagnosis to first MTX prescription was 0.66 (IQR 0.26–1.80) years. Post hoc division of the material showed a time lapse of 0.59 (IQR 0.24–1.18) years in the period 1998–2004 and 0.92 (0.31–4.41) years in the period 2005–2012 (Table 1).

Table 2 presents the crude determinants of receiving only one or receiving two or more MTX prescriptions. In general those with a comorbid condition were less likely to file more than one prescription of MTX. However, in some cases the differences were small in absolute terms (e.g., 85% versus 84% for prior prednisolone use or not) although the difference attained statistical significance due to the large number of exposed subjects.

**Figure 2:**
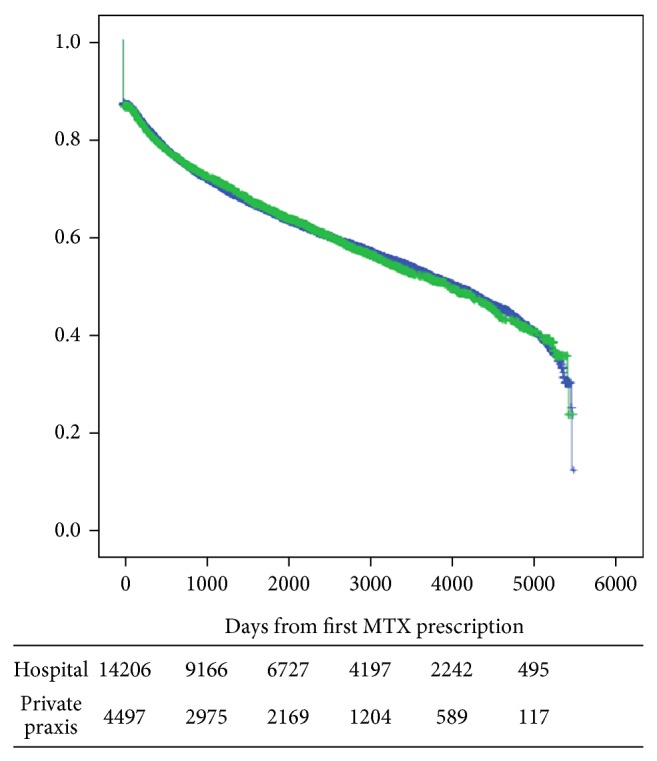
Adherence to MTX. Use of 7.5 mg per week stratified by whether the patient was seen by a practicing specialist or not. Days from first prescription.

**Figure 3:**
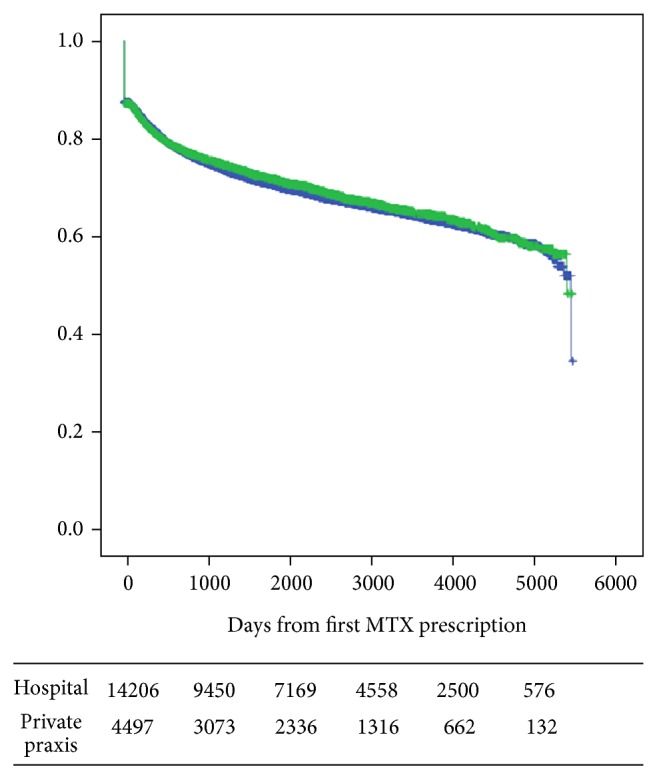
Adherence to MTX. Use of 5 mg per week stratified by whether the patient was seen by a practicing specialist or not. Days from first prescription.

Figure 2 (including all prescriptions) and Figure 3 (only considering those who filed two or more MTX prescriptions) show the Kaplan-Meier plots of adherence to MTX stratified by whether they were seen by practising specialists in rheumatology or not. No difference in adherence to MTX was present between those managed in private practice (1,925 (IQR 467–3,056) days) versus 1,892 (IQR 452–3,316) days for patients treated in hospital. In those who filed more than one MTX prescription, the mean adherence time for 7.5 mg MTX per week was 2,245 (IQR 986–3,407) days.

Table 3 shows the crude and adjusted hazard rates (HR) of using less than 5 mg of MTX per week or 7.5 mg of MTX per week, respectively. The adjustment in the Cox proportional hazard regression included all confounders shown. Adjustment only changed the estimates little. In general female gender, T2D with complications, COPD, atherosclerosis, mild liver disease, and kidney disease all increased the likelihood of nonadherence to both 5 and 7.5 mg MTX per week in the multiple adjusted analysis. Increasing age decreased the likelihood of nonadherence (i.e., elderly were more compliant with MTX). Increasing time span between diagnosis and first prescription of MTX increased the likelihood of nonadherence. Being seen by a rheumatologist in private practice did not differ from hospital follow-up in terms of adherence to MTX.

## 4. Discussion

The present study demonstrated that, after an initial loss of adherence, the remainder Danish RA patients slowly but steadily dropped out of treatment over the following years. “Half-life” on MTX in patients with more than one prescription was, however, over 4000 days, provided an acceptance of 7.5 mg MTX per week as compliant, corresponding to the lower limit of recommended dose. These results are in some contrast with the notion of a very low adherence to MTX, while they add a time perspective to previous indications [13] of the development of nonadherence [8, 9]. The even better adherence to 5 mg MTX per week seems to confirm our hypothesis that patients taper their dosage of MTX to a rather low level, while they do not fully withdraw from this treatment.

The adherence to MTX was associated with general health as demonstrated by the significantly higher drop-out rate in patients with comorbid conditions, while this development may also reflect a modulation of the treating physician due to comorbidities. Nevertheless, the elderly were more compliant with MTX than younger RA patients, which seems to confirm our immediate expectations that in general the elderly do what their treating physician tells them more often than younger subjects.

The main part of the participants, some 85%, had received prednisolone for the RA, while interestingly use of prednisolone at baseline did not influence the adherence to MTX to any clinically significant extent. There was delay of more than a half year on average from diagnosis to the first prescription of MTX, which may reflect a Danish tradition with prednisolone being used more than recommended by current guidelines [5]. This diversion from standard may potentially affect the adherence as demonstrated by the lower retention rate with increasing time between diagnosis and MTX prescription.

### 4.1. Limitations

Our study group was defined by a diagnosis of RA from the hospital register. Thus, patients having never been admitted to hospital and only having received treatment in rheumatology practice were not included. However, we found no difference in adherence between patients having prescriptions of MTX at a hospital outpatients clinic as compared to patients being followed up by a practising rheumatologist. In a hospital setting, Danish RA patients are subjected to more frequent changes in treating physician than in rheumatology practice, which in Denmark is run by only one specialist. This observation also leads to a conclusion that Danish RA patients comply with medication due to tradition rather than personal contact with a specific doctor. As a confounder to this statement, some patients may have initiated their treatment in one or other of these institutions, while continuing therapy in the opposite.

The present study assumed that the dataset from The National Pharmacological Database reflected actual use of MTX, while no interviews or other indications of actual ingestion of MTX were obtained. The data, however, add to the experiences from other countries, but in our case based on robust prescription data from high quality Danish registers. A possible development toward earlier prescriptions of MTX due to changes in guidelines may be expected in the future, but not yet detectable in a retrospective cohort as in the present study. Unfortunately, registration of biological medications is not given in the national, mandatory databases used in the study, and thus no data of a possible influence of such medication on MTX use were available.

Associations between adherence to MTX and clinical impact on disease activity could not be detected in the official databases and no systematic registration is available of, for example, disease activity scores including joint evaluations.

### 4.2. Perspectives

The present data suggest that, in Denmark, only about half of the RA patients are given the recommended first-line treatment with MTX. Also, on average the first prescription of MTX is delayed for more than half a year after the diagnosis of RA. The possible consequences would seem to be a risk of erosions developing during this lag-phase. With notion of effective and targeted therapy, especially with usage of MTX [6, 7], this local tradition would need to be changed.

## 5. Conclusions

Danish RA patients receive MTX for their disease only in little less than half of the cases. This result is the same whether treatment is monitored by a hospital clinic or practicing rheumatologist. Once on MTX, the majority of patients adhere for many years to the treatment but taper the dosage to rather low levels. Adherence to MTX therapy decreases with time lapse between diagnosis and prescription. All these results suggest that a more meticulous instruction and campaign towards early TMX use is relevant among both rheumatologists and patients.

## Figures and Tables

**Table 1:** Characteristics of RA patients at the time of first MTX prescription.

Variables	Number
Age (years ± SD)	59.8 ± 14.4
Men	5,221 (28%)
Women	13,482 (72%)
AMI	774 (4%)
Cerebrovascular disease	991 (5%)
Dementia	80 (0.4%)
Complications to T1D	183 (1%)
Complications to T2D	279 (2%)
T1DM	322 (2%)
T2DM	734 (4%)
Hemiplegia	30 (0.2%)
Heart failure	687 (4%)
Peripheral vascular disease	694 (4%)
Chronic lung disease	1,562 (8%)
Leukemia	29 (0.2%)
Lymphoma	99 (0.5%)
Metastases	98 (0.5%)
Mild liver disease	204 (1%)
Moderate to severe liver disease	29 (0.2%)
Kidney disease	328 (2%)
Cancer	476 (3%)
Ulcer	1,207 (7%)
NSAID within the last year	13,415 (72%)
Prednisolone/prednisone within the last year	9,052 (48%)
Ever prior use of prednisolone/prednisone	11,444 (61%)
Ever seen by private practicing rheumatologist	4,497 (24%)
Time from diagnosis to first MTX prescription (years)	0.66 (IQR 0.26–1.80)

T1DM: type 1 diabetes mellitus.

T2DM: type 2 diabetes mellitus.

**Table 2:** Characteristics of patients filing only one or filing more than one MTX prescription: predictors of filing only one or filing two or more MTX prescriptions.

Variables	One MTX prescription	Two or more MTX prescriptions	*P* value (Pearson chi-square test, 2-sided)
≤50 years	679 (15%)	3,849 (85%)	<0.001
51–60 years	648 (15%)	3,672 (85%)	
61–70 years	631 (13%)	4,223 (87%)	
>70 years	950 (19%)	4,053 (81%)	
Men	784 (15%)	4,447 (85%)	0.089
Women	2,161 (16%)	11,350 (84%)	
No prior use of prednisolone/prednisone	1,053 (15%)	6,206 (85%)	0.002
Prior use of prednisolone/prednisone	1,853 (16%)	9,591 (84%)	
No AMI	2,741 (15%)	15,188 (85%)	<0.001
AMI	165 (21%)	609 (79%)	
No cerebrovascular disease	2,697 (15%)	15,015 (85%)	<0.001
Cerebrovascular disease	209 (21%)	782 (79%)	
No dementia	2,884 (15%)	15,739 (85%)	0.003
Dementia	22 (27%)	58 (73%)	
No complications to T1D	2,860 (15%)	15,660 (85%)	<0.001
Complications to T1D	46 (25%)	137 (75%)	
No complications to T2D	2,834 (15%)	15,590 (85%)	<0.001
Complications to T2D	72 (26%)	207 (74%)	
No T1D	2,830 (15%)	15,551 (85%)	<0.001
T1D	76 (24%)	246 (76%)	
No T2D	2,751 (15%)	15,218 (85%)	<0.001
T2D	155 (21%)	579 (79%)	
No hemiplegia	2,898 (15%)	15,775 (85%)	0.092
Hemiplegia	8 (27%)	22 (73%)	
No heart failure	2,735 (15%)	15,281 (85%)	<0.001
Heart failure	171 (25%)	516 (75%)	
No peripheral vascular disease	2,741 (15%)	15,268 (85%)	<0.001
Peripheral vascular disease	165 (24%)	529 (76%)	
No chronic lung disease	2,553 (15%)	14,588 (85%)	<0.001
Chronic lung disease	353 (23%)	1,209 (77%)	
No leukemia	2,901 (15%)	15,773 (85%)	0.800
Leukemia	5 (17%)	24 (83%)	
No lymphoma	2,886 (15%)	15,718 (85%)	0.199
Lymphoma	20 (20%)	79 (80%)	
No metastases	2,880 (15%)	15,725 (85%)	0.003
Metastases	26 (26%)	72 (74%)	
No mild liver disease	2,852 (15%)	15,647 (85%)	<0.001
Mild liver disease	54 (26%)	150 (74%)	
No moderate to severe liver disease	2,903 (15%)	15,771 (85%)	0.440
Moderate to severe liver disease	3 (10%)	26 (90%)	
No kidney disease	2,816 (15%)	15,560 (85%)	<0.001
Kidney disease	90 (27%)	237 (73%)	
No cancer	2,813 (15%)	15,414 (85%)	0.015
Cancer	93 (19%)	383 (81%)	
No ulcer	2,664 (15%)	14,832 (85%)	<0.001
Ulcer	242 (20%)	965 (80%)	

**Table 3:** Risk of nonadherence to MTX: Cox proportional hazard model; crude and multiply adjusted hazard rates (HR) and 95% confidence intervals (CI).

Variables at baseline	5 mg per week	7.5 mg per week	5 mg per week^#^	7.5 mg per week^#^
	Crude OR (95% CI)	Crude OR (95% CI)	HR (95% CI)	HR (95% CI)
Gender (F/M)	1.21 (1.13–1.30)^*^	1.18 (1.11–1.26)^*^	1.12 (1.06–1.19)^*^	1.07 (1.01–1.12)^*^
NSAID within last year	1.09 (1.01–1.16)^*^	1.08 (1.01–1.15)^*^	1.03 (0.97–1.09)	1.01 (0.96–1.06)
Prednisolone within last year	0.91 (0.86–0.97)^*^	0.93 (0.88–0.98)^*^	0.99 (0.94–1.04)	1.00 (0.96–1.04)
Biological antirheumatic therapy	0.74 (0.24–2.32)	1.48 (0.54–4.07)	0.65 (0.24–1.73)	1.04 (0.52–2.09)
AMI	0.90 (0.77–1.05)	0.85 (0.73–0.98)^*^	1.07 (0.93–1.22)	1.01 (0.90–1.14)
Cerebrovascular events	0.94 (0.82–1.08)	0.95 (0.84–1.09)	1.11 (0.99–1.25)	1.14 (1.03–1.26)^*^
Dementia	0.77 (0.47–1.26)	1.01 (0.65–1.57)	1.14 (0.75–1.74)	1.46 (1.05–2.02)^*^
T1D with complications	1.07 (0.79–1.45)	1.01 (0.75–1.35)	0.97 (0.71–1.33)	0.92 (0.69–1.21)
T2D with complications	1.29 (1.01–1.65)^*^	1.26 (0.99–1.59)	1.34 (1.07–1.68)^*^	1.30 (1.06–1.58)^*^
T1D	1.06 (0.84–1.33)	1.05 (0.84–1.31)	0.93 (0.72–1.19)	0.96 (0.78–1.19)
T2D	1.05 (0.90–1.23)	1.08 (0.93–1.25)	1.06 (0.91–1.24)	1.10 (0.96–1.25)
Hemiplegia	1.36 (0.66–2.81)	1.14 (0.56–2.33)	1.45 (0.82–2.57)	1.33 (0.78–2.24)
Heart failure	0.91 (0.77–1.08)	0.94 (0.81–1.10)	1.12 (0.97–1.29)	1.20 (1.06–1.36)^*^
Atherosclerosis	0.93 (0.79–1.09)	1.01 (0.86–1.17)	1.12 (0.97–1.28)	1.23 (1.10–1.39)^*^
COPD	1.10 (0.98–1.22)	1.03 (0.93–1.14)	1.23 (1.12–1.35)^*^	1.21 (1.11–1.31)^*^
Leukemia	0.65 (0.28–1.51)	1.06 (0.51–2.20)	0.85 (0.41–1.79)	1.37 (0.80–2.37)
Lymphoma	0.93 (0.61–1.42)	0.96 (0.64–1.43)	1.03 (0.72–1.47)	1.05 (0.77–1.42)
Metastatic malignancy	0.62 (0.39–0.99)^*^	0.60 (0.39–0.92)^*^	0.85 (0.57–1.29)	0.90 (0.63–1.28)
Mild liver disease	1.35 (1.02–1.78)^*^	1.25 (0.95–1.65)	1.30 (1.05–1.63)^*^	1.28 (1.05–1.56)^*^
Moderate liver disease	0.65 (0.28–1.51)	0.68 (0.32–1.46)	0.70 (0.33–1.47)	0.76 (0.41–1.42)
Kidney disease	1.39 (1.11–1.73)^*^	1.30 (1.04–1.61)^*^	1.35 (1.14–1.61)^*^	1.31 (1.12–1.53)^*^
Solid cancer	0.91 (0.75–1.11)	0.85 (0.71–1.03)	1.05 (0.89–1.24)	1.00 (0.86–1.16)
Ulcer disease	0.94 (0.83–1.06)	0.96 (0.85–1.07)	1.07 (0.96–1.18)	1.08 (0.98–1.18)
Age				
<50 years	1 (reference)	1 (reference)	1 (reference)	1 (reference)
50–59 years	0.79 (0.72–0.86)^*^	0.76 (0.70–0.82)^*^	0.84 (0.79–0.90)^*^	0.83 (0.78–0.88)^*^
60–69 years	0.59 (0.55–0.65)^*^	0.58 (0.53–0.63)^*^	0.70 (0.66–0.76)^*^	0.73 (0.69–0.78)^*^
≥70 years	0.59 (0.54–0.64)^*^	0.61 (0.56–0.66)^*^	0.77 (0.72–0.83)^*^	0.89 (0.83–0.95)^*^
Private practising rheumatologist	0.95 (0.89–1.03)	0.99 (0.92–1.06)	0.97 (0.92–1.03)	1.02 (0.97–1.07)
Difference from diagnosis to MTX				
<0.5 years	1 (reference)	1 (reference)	1 (reference)	1 (reference)
0.5–0.99 years	0.69 (0.64–0.75)^*^	0.74 (0.69–0.80)^*^	0.74 (0.69–0.79)^*^	0.80 (0.76–0.85)^*^
≥1 year	1.07 (1.00–1.15)	1.02 (0.96–1.09)	1.17 (1.11–1.24)^*^	1.21 (1.15–1.27)^*^

F: female; M: male; AIDS: acquired immune-deficiency syndrome; AMI: acute myocardial infarction; T1D: type 1 diabetes; COPD: chronic obstructive pulmonary disease.

^#^Mutually adjusted for the other variables in the table (Cox proportional hazard regression).

^*^ *P* < 0.05.
